# Stability of chronotype over a 7‐year follow‐up period and its association with severity of depressive and anxiety symptoms

**DOI:** 10.1002/da.22995

**Published:** 2020-02-17

**Authors:** Stella J. M. Druiven, Johanna H. M. Hovenkamp‐Hermelink, Stefan E. Knapen, Jeanine Kamphuis, Benno C. M. Haarman, Brenda W. J. H. Penninx, Niki Antypa, Ybe Meesters, Robert A. Schoevers, Harriëtte Riese

**Affiliations:** ^1^ Department of Psychiatry, Interdisciplinary Center Psychopathology and Emotion regulation (ICPE), University Medical Center Groningen University of Groningen Groningen The Netherlands; ^2^ Department of Psychiatry, Research School of Behavioral and Cognitive Neurosciences (BCN), University Medical Center Groningen University of Groningen Groningen The Netherlands; ^3^ Department of Psychiatry, EMGO+ Institute VU University Medical Center Amsterdam The Netherlands; ^4^ Department of Clinical Psychology, Institute of Psychology Leiden University Leiden The Netherlands

**Keywords:** anxiety symptoms, chronotype, depressive symptoms, test–retest

## Abstract

**Background:**

Chronotype is an individual's preferred timing of sleep and activity, and is often referred to as a later chronotype (or evening‐type) or an earlier chronotype (or morning‐type). Having an evening chronotype is associated with more severe depressive and anxiety symptoms. Based on these findings it is has been suggested that chronotype is a stable construct associated with vulnerability to develop depressive or anxiety disorders. To examine this, we test the stability of chronotype over 7 years, and its longitudinal association with the change in severity of depressive and anxiety symptoms.

**Methods:**

Data of 1,417 participants with a depressive and/or anxiety disorder diagnosis and healthy controls assessed at the 2 and 9‐year follow‐up waves of the Netherlands Study of depression and anxiety were used. Chronotype was assessed with the Munich chronotype questionnaire. Severity of depressive and anxiety symptoms were assessed with the inventory of depressive symptomatology and Beck anxiety inventory.

**Results:**

Chronotype was found to be moderately stable (*r* = 0.53) and on average advanced (i.e., became earlier) with 10.8 min over 7 years (*p* < .001). Controlling for possible confounders, a decrease in severity of depressive symptoms was associated with an advance in chronotype (*B* = 0.008, *p* = .003). A change in severity of anxiety symptoms was not associated with a change in chronotype.

**Conclusion:**

Chronotype was found to be a stable, trait‐like construct with only a minor level advance over a period of 7 years. The change in chronotype was associated with a change in severity of depressive, but not anxiety, symptoms.

## 1 INTRODUCTION

Chronotype is an individual's preferred timing of sleep and activity, and is often referred to as a later chronotype (or evening‐type) or an earlier chronotype (or morning‐type). Being an evening‐type has been cross‐sectionally associated with more severe depressive and anxiety symptoms (Hsu, Shur‐Fen Gau, Shang, Chiu, & Lee, 2012; Kitamura et al., 2010), and having a current depressive disorder diagnosis (Drennan, Klauber, Kripke, & Goyette, 1991). The authors of the latter study suggested chronotype to be a trait‐like, and thus a relatively stable, construct, with being an evening‐type indicating a vulnerability for developing a depression. However, as no repeated measurements of chronotype were obtained in the study of Drennan, prospective conclusions cannot be drawn from these data. In current literature, there are only four studies that addressed the stability of chronotype by using repeated measurements (Broms et al., 2014; Caci, Nadalet, Staccini, Myquel, & Boyer, 2000; Koskenvuo, Hublin, Partinen, Heikkilä, & Kaprio, 2007; Maukonen, Kanerva, Partonen, & Männistö, 2019).

Caci and colleagues studied the stability of the French version of the composite scale of morningness (CSM) (Caci et al., 2000). The CSM was filled‐out by 60 healthy, young adults on two occasions over a 13‐month period, and the mean scores did not differ between the two occasions. Koskenvuo et al assessed chronotype in 190 healthy participants from a twin study by twice asking a single question: *“Will you try to estimate to what extent you're being a morning or an evening person?”* (Koskenvuo et al., 2007). At baseline and at 6‐year follow‐up, 63% of the participants reported the same chronotype; 68% of all morning‐types reported being a morning‐type at both occasions, and 44% consistently reported being an evening‐type. Using the same question, Broms et al. (2014) reported similar results in a 23 year follow‐up study in a group of 567 male adults. At baseline and follow‐up, 65% of the morning‐type participants reported to consider themselves being a morning‐type at both assessments, and 34% twice reported to be an evening‐type. While it was concluded by Koskenvuo that chronotype was stable over time, the lower percentage of evening‐types reporting the same chronotype over time in both studies could indicate that evening‐type can be subject to change (Broms et al., 2014; Koskenvuo et al., 2007). A rather stable chronotype, assessed with a shortened version of the morningness–eveningness questionnaire (MEQ, Horne & Ostberg, 1976), can be assumed in a 7‐year follow‐up study of Maukonen et al. (2019). At baseline participants were categorized as morning‐types (*n* = 552), intermediate types (*n* = 433) or evening‐types (*n* = 112). At follow‐up (*n* = 919); there were four baseline evening‐types classified as morning‐types and five baseline morning‐types classified as evening‐types at follow‐up.

Moreover, the long‐term stability of chronotype and its association to fluctuations in severity of depressive and anxiety symptoms is largely unexplored. To the best of our knowledge, only one study addressed this question (Müller et al., 2015). They found that the preference for sleep timing in depressive patients, as assessed with the MEQ, was highly correlated (*r* = 0.82, *p* < .001) over the course of a hospitalization period (mean stay: 48.6 days), despite a significant improvement in patient's depressive symptoms. However, the time period studied might have been too short to pick up subsequent changes in chronotype. In addition, we showed in our previous work that chronotype was not predictive of a 4‐year persistent diagnosis of depressive and anxiety disorder (Druiven et al., 2019). This finding could support the assumption that chronotype is not a trait‐like construct in the way it was suggested by Drennan et al. (1991): If chronotype would be a trait that is associated with having more depressive or anxiety symptoms and having a diagnosis of depressive and/or anxiety disorder, it would also most likely predict a diagnosis in the future. Another study indeed showed that evening‐type was predictive of an increase of depressive symptoms and a depressive diagnosis 1 year later in a group of adolescents (Haraden, Mullin, & Hankin, 2017). These conflicting results illustrate the need for more longitudinal studies on the associated changes in severity of depressive and anxiety symptoms and chronotype.

In the current study we aim to: (a) Test the 7‐year stability of chronotype, and (b) analyze whether a longitudinal association exists between a change in severity of depression and anxiety symptoms and change in chronotype.

## 2 METHODS

### 2.1 Study sample

**Figure 1:**
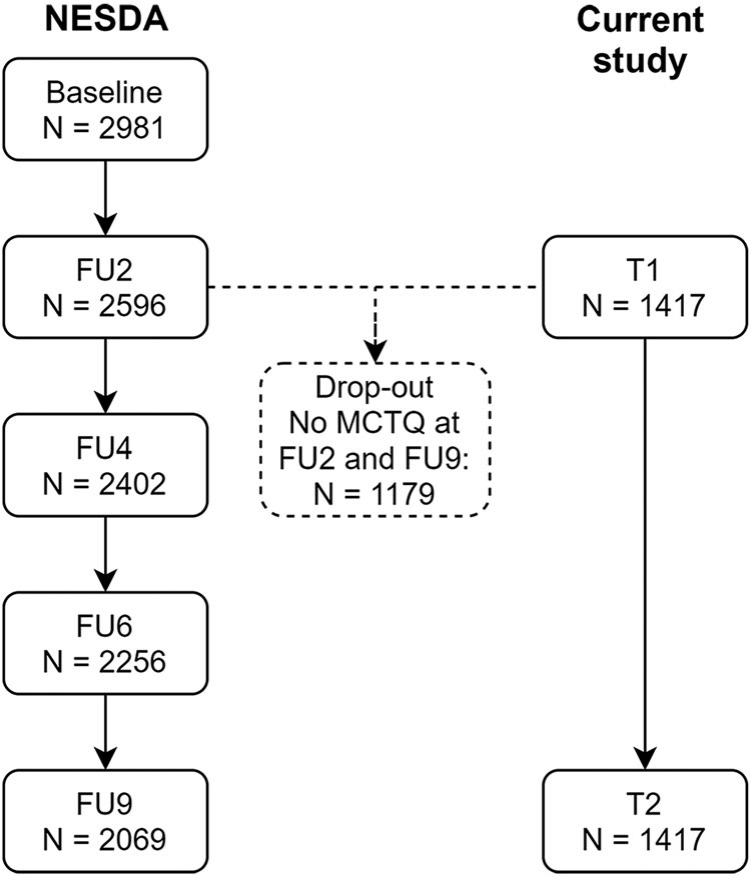
Flow‐chart from the 2‐ (T1) and 9‐year (T2) follow‐up of NESDA of those included in the current study. Participants with incomplete chronotype assessments (Munich chronotype questionnaire) at one or both time points were excluded. FU2: 2‐year follow‐up; FU4: 4‐year follow‐up; FU6: 6‐year follow‐up; FU9: 9‐year follow‐up. MCTQ, Munich chronotype questionnaire; NESDA, the Netherlands study of depression and anxiety

Data from the Netherlands study of depression and anxiety (NESDA) were used (Penninx et al., 2008). NESDA is a Dutch ongoing study to the longitudinal course of depressive and anxiety disorder. A total of 2,981 participants were included at baseline of which 2,329 participants with a current or past diagnosis of depressive and/or anxiety disorder and 652 healthy controls. Patients with a diagnosis for psychotic disorder, obsessive compulsive disorder, bipolar disorder, or severe addiction disorder were not included. All participants (age 18–65 years) were included through mental healthcare organizations, the general community and primary care. A detailed method and rationale is described elsewhere (Penninx et al., 2008). Baseline inclusion began in 2004 and ended in 2007. The face‐to‐face follow‐up assessments after 2, 4, 6, and 9 years had relatively high response rates with 87% (*n* = 2,596), 81% (*n* = 2,402), 76% (*n* = 2,256), and 69% (*n* = 2,069), respectively (van Eeden et al., 2019). The ethical committees of participating universities approved the study protocol and participants provided written informed consent. As shown in Figure 1, the current study used data from the 2‐year (which will be called T1 from this point forward) and 9‐year follow‐up (T2) as only these follow‐up points included chronotype assessments.

### 2.2 Chronotype

The Munich chronotype questionnaire (MCTQ) was used to assess chronotype (Roenneberg, Wirz‐Justice, & Merrow, 2003). The MCTQ is a self‐report questionnaire composed of questions about the actual timing of sleep on workdays and free days separately. From these times, the Midsleep on free days (MSF) can be calculated which is the midpoint between sleep onset and offset on free days. Sleep onset at T1 was calculated by adding the answers of two questions from the MCTQ: ‘I go to bed at.’ and “Time needed to fall asleep (minutes).” At T2 the MCTQ was slightly altered in a way that another question was asked after ‘I go to bed at.’ which was: ‘I decide to go to sleep at. (i.e., I close my eyes at).’ Because this question was not included at T1, participants at T1 may have answered the question ‘I go to bed at.’ as the moment that they closed their eyes. In the case of someone with stable sleep timing over 7 years, this change in questions could have caused chronotype to falsely appear earlier at T1 compared with T2. Therefore, sleep onset at T2 was calculated by adding the time needed to fall asleep by the latest time of the questions ‘I go to bed at.’ and ‘I decide to go to sleep at.’ However, as a robustness check, sleep onset was calculated in two alternative ways using the separate questions and adding the time needed to fall asleep. The full procedure and the results after repeating the planned statistics (described below) are described in the supplemental materials (Tables S2,S3). In short, results were highly similar and thus conclusions drawn from the results described in the main manuscript should be considered as robust.

Some individuals can experience oversleep on free days because of sleep deprivation during the week due to work hours. For these persons, the MSF measure is corrected by subtracting from MSF half of the difference between sleep duration on free days and average weekly sleep duration (Roenneberg, Allebrandt, Merrow, & Vetter, 2012). The corrected MSF (MSFsc), used in this study, is a validated measure for chronotype (Zavada, Gordijn, Beersma, Daan, & Roenneberg, 2005). MSFsc reflects the number of hours after midnight, for example, a MSFsc of 1.5 corresponds to 01:30 a.m. As a result, higher scores of MSFsc reflect a later chronotype and lower MSFsc scores reflect an earlier chronotype.

### 2.3 Measures

#### 2.3.1 Depressive and anxiety severity

Severity of depressive and anxiety symptoms were assessed at T1 and T2 using the inventory of depressive symptomatology—self report (IDS‐SR) and the Beck anxiety inventory (BAI). The IDS consists of 28 questions including DSM‐IV criteria for major depressive disorder and associated symptoms such as anxiety and irritability and atypical and melancholic symptoms (Trivedi et al., 2004). Each question is scored between 0 and 3 reflecting the severity of symptoms during the past week, which results in a sum score of 0–84, with higher scores indicating higher depression severity. The BAI is a self‐report instrument which consists of 21 items (Beck, Epstein, Brown, & Steer, 1988). Each item is scored from 0 to 3 reflecting the experienced of symptoms over the past week. The sum score can range from 0 to 63, with higher scores corresponding with increasing anxiety severity.

#### 2.3.2 Depressive and anxiety diagnosis

For descriptive reasons, the one‐month diagnosis (i.e., diagnosis present in the month before the interview) of a depressive (MDD and dysthymia) or anxiety (panic disorder, social phobia, generalized anxiety disorder, and agoraphobia) disorder was assessed at T1 and T2 with the composite international diagnostic interview (CIDI), version 2.1 (World Health Organization, 1997). The CIDI is a validated instrument created in accordance with DSM‐IV criteria (Wittchen, 1994).

#### 2.3.3 Insomnia

Because of the association between insomnia and chronotype and severity of depressive and anxiety symptoms, the severity of insomnia was included as a covariate in this study (Alvaro, Roberts, & Harris, 2014). In NESDA, it was assessed at both time‐points by the Women's health initiative insomnia rating scale. It is a self‐report questionnaire including five items about different aspects of sleep in the past 4 weeks (Levine et al., 2003). The scores per item can range between 0 and 4 and the sum score ranges between 0 and 20.

#### 2.3.4 Sociodemographic factors

The analyses will be controlled for possible confounding variables that may influence both chronotype and symptoms, such as age, sex, having children in the household and employment status (Bjelland et al., 2008; Díaz‐Morales & Pilar Sánchez‐López, 2008). All sociodemographic factors were assessed at T1 and T2. Employment status (yes/no) and having children in the household (yes/no) were obtained by self‐report.

### 2.4 Statistical analyses

Data from the MCTQ of 1,417 participants were available to calculate chronotype. However, participants with missing data at one of the assessments were included in the main analyses (insomnia T1: *N* = 1, 0.07%; employment status T1: *N* = 26, 1.83%), as the planned statistical analysis could adequately handle missing data. For the descriptive statistics, chronotype, the severity of depressive and anxiety symptoms and sociodemographic factors were compared between the two time‐points. A McNemar test was used to compare dichotomous and categorical variables (sex, employment status, and children in the household) between T1 and T2. A the Wilcoxon signed rank test was used for comparing continuous variables (severity of depressive symptoms, severity of anxiety symptoms, severity of insomnia, age) between T1 and T2.

The stability of chronotype between T1 and T2 was analyzed by comparing mean scores using a the Wilcoxon signed rank test, and by calculating the correlation coefficient as a Spearman's correlation. For this, correlations below 0.3 were considered small, between 0.3 and 0.5 medium and 0.5 or higher large (Cohen, 1988).

For testing the longitudinal association between the changes in severity of depressive and anxiety symptoms and the change in chronotype generalized estimating equation (GEE) analyses were used. The GEE analysis is a form of regression analysis that corrects for within‐subject correlations. The technique generates a regression coefficient that reflects the longitudinal association between the change in independent variable (symptom severity) and dependent variable (chronotype; Twisk & Vente, 2000). First, a GEE analysis was conducted with entering only depressive symptoms (Model 1), and only anxiety symptoms (Model 2) as independent variables. Second, both depressive and anxiety symptoms were added as independent variables (Model 3). Third, sex, children in the household, employment status, and severity of insomnia were added as independent variables to Model 3 (Model 4). Finally, all covariates were entered (severity of depressive symptoms, severity of anxiety symptoms, severity of insomnia, age, sex, children in the household, employment status) in the model (Model 5). For the GEE, sex was treated as time‐independent variable, whereas severity of depressive and anxiety symptoms, severity of insomnia, age, children in the household and employment status were treated as time‐dependent variables.

To check for multicollinearity for all variables, the variance inflation factor (VIF) and Spearman correlation were calculated before performing the GEE analyses. VIF values above 10 and Spearman correlations above 0.80 were considered as indication of severe collinearity (Dormann et al., 2013; Field, 2009). Data were analyzed in the Statistical Package for Social Science (IBM SPSS, Chicago, IL version 23.0 for Windows). *p *< .05 were considered statistically significant.

## 3 RESULTS

### 3.1 Descriptives

The sociodemographic, lifestyle, and clinical factors of the sample are given in Table 1. At T2, the participants were older, had more years of education, more frequently had a child in their household, were less often employed, and less participants had a diagnosis of a depressive and/or an anxiety disorder. Severity of insomnia symptoms, and severity of depressive symptoms did not differ between T1 and T2, whereas severity of anxiety symptoms significantly decreased.

**Table 1:** Sociodemographic, lifestyle, and clinical factors assessed at T1 and T2 (*N* = 1,417)

Characteristics	T1	T2	*p* ^a^
Sex, n (%) women	935 (65.98)	935 (65.98)	
Age, year, M (*SD*)	42.46 (12.78)	49.54 (12.79)	**<.001**
Child in household, n (%), yes	487 (34.37)	542 (38.25)	**.004**
Employment status, n (%), yes	1,084 (76.50)	998 (70.43)	**<.001**
Severity of depressive symptoms, M (*SD*)	13.80 (10.84)	13.70 (10.87)	.482
Severity of anxiety symptoms, M (*SD*)	7.43 (7.77)	7.05 (7.71)	**.010**
Insomnia, M (*SD*)	6.79 (4.43)	6.90 (4.57)	.584
Depressive disorder diagnosis CIDI,^b^ n (%), yes	179 (12.63)	139 (9.81)	**.009**
Anxiety disorder diagnosis CIDI,^b^ n (%), yes	263 (18.56)	200 (14.11)	**<.001**
Chronotype in MSFsc, M (*SD*)	3.95 (0.97)	3.77 (0.96)	**<.001**

*Note*: Bold values indicate *p* < .05.

Abbreviations: CIDI, composite international diagnostic interview; MSFsc: MidSleep on free days sleep corrected; *SD*, standard deviation.

^a^ Wilcoxon signed rank tests were used to compare the continuous characteristics (age, depressive symptoms, anxiety symptoms, insomnia symptoms), McNemar tests were used to compare dichotomous characteristics (child in household, employment status, depressive disorder diagnosis, anxiety disorder diagnosis).

^b^ The 1‐month CIDI diagnoses were used (diagnosis present in the month before the assessment); T1: NESDA's 2‐year follow‐up, T2: NESDA's 9‐year follow‐up, MSFsc: MidSleep on Free days sleep corrected, CIDI: Composite International Diagnostic Interview.

For all variables, the VIF values were between 1.01 and 3.01. The correlations between the variables are given in the supplemental material (Table S1), the highest correlation was 0.79 between depressive and anxiety symptoms. As was defined in the method section, the variables were considered not to suffer from severe collinearity and were used in the GEE analyses.

### 3.2 Stability of chronotype

**Figure 2:**
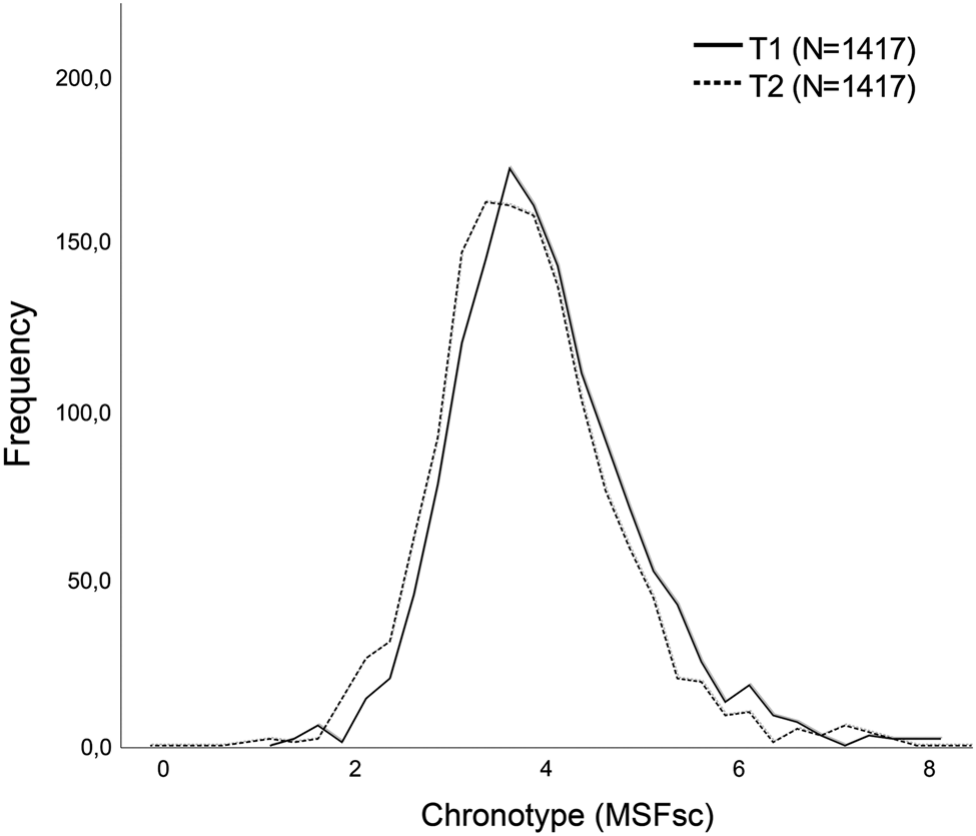
Frequency distribution of chronotype, MSFsc (hours), at T1 and T2. MSFsc, MidSleep on free days sleep corrected

Mean MSFsc at T2 (Table 1) was 10.8 min earlier compared to T1. This means that participants reported going to bed/fall asleep earlier at T2 (Figure 2). The test–retest correlation of MSFsc was 0.53 (*p* < .001), which is considered a large correlation.

### 3.3 GEE analyses

The results of the GEE analyses are given in Table 2. Model 1 showed that a decrease in severity of depressive symptoms is associated with a decrease in MSFsc (which corresponds with an earlier chronotype). This result should be interpreted as follows: a decrease of 1 unit of severity of depressive symptoms (a decrease of 1.0 in IDS score) is associated with a decrease of 0.006 of MSFsc, which corresponds to an advance of 0.36 min. In Model 2, there is an association between a decrease in severity of anxiety symptoms and a decrease in MSFsc. When entered simultaneously (Model 3), there is no association between change in severity of depressive and anxiety symptoms and a change in MSFsc. Both Models 4 and 5, where potential confounders were additionally entered as predictors, again showed that a decrease of 1 unit of severity of depressive symptoms was associated with a decrease of 0.008 MSFsc (advance of 0.48 min). However, no association was found between a change in severity of anxiety symptoms and a change in MSFsc in latter models (Models 4 and 5).

**Table 2:** Results of the GEE analyses: Longitudinal associations between change in severity of depressive and anxiety symptoms and change in chronotype (MSFsc) analyzed by generalized estimating equations (*N* = 1417)

	MSFsc		
	B	95% CI	*p*
Model 1			
Depressive symptoms	0.006	0.002–0.009	**.003**
Model 2			
Anxiety symptoms	0.006	0.000–0.011	**.021**
Model 3			
Depressive symptoms	0.005	0.000–0.010	.066
Anxiety symptoms	0.001	−0.006–0.009	.753
Model 4^a^			
Depressive symptoms	0.008	0.002–0.013	**.004**
Anxiety symptoms	0.001	−0.007–0.008	.829
Model 5^b^			
Depressive symptoms	0.008	0.002–0.013	**.004**
Anxiety symptoms	0.000	−0.007–0.007	.995

*Note*: Bold values indicate *p* < .05.

Abbreviations: GEE, generalized estimating equation; MSFsc, MidSleep on free days sleep corrected.

^a^ Model 4: Additionally adjusted for sex, children in household, employment, insomnia level.

^b^ Model 5: Additionally adjusted for age.

### 3.4 Posthoc‐analyses

To interpret the findings of our GEE analyses in posthoc analyses, participants were categorized into having an advanced (became earlier), delayed (became later) or stable chronotype. Categorization was based on a participant's personal change in MSFsc between T1 and T2. Although a golden standard is lacking, differences in MSFsc have been reported between healthy controls and patients with a depressive disorder of less than 30 min (Knapen et al., 2018), or around 30 min (Morelatto De Souza, Paz, & Hidalgo, 2014). We considered MSFsc to be stable over time if the difference in MSFsc was less than 30 min (i.e., ΔMSFsc < 0.5, *N* = 665 [46.9%]), and instable if there was an advance (MSFsc T1 >T2, *N* = 473 [33.4%]) or a delay (MSFsc T1 <T2, *N* = 279 [19.7%]) of more than 30 min. In the supplementary material figure S1 a graph of this categorization is shown, which was validated by testing differences in mean chronotype between time points (results are given in supplemental material table S4 together with descriptives of sociodemographic, lifestyle and clinical factors per group).

Next, the GEE analyses were repeated with these groups (advanced, stable and delayed chronotype) using Model 1–5 as described in the method section. Results are given in Table 3. The stable and delayed chronotype groups did not show any associations between a change in depressive and anxiety symptom severity and a change in MSFsc. However, in line with the findings of the main GEE analyses, the advanced chronotype group showed an association between a decrease in severity of depressive symptoms and a decrease in MSFsc in Model 1, where a decrease of 1 unit of depressive symptoms was associated with a decrease of 0.009 MSFsc (i.e., an advance of 0.54 min). Model 2 showed an association between a decrease in severity of anxiety symptoms and a decrease in MSFsc. This association was no longer significant when severity of depressive and anxiety symptoms were entered simultaneously in the model (Model 3). Models 4 and 5, where potential confounders were added to the model, showed an association between a decrease in severity of depressive symptoms and a decrease in MSFsc. However, there was no association between a change in severity of anxiety symptoms and a change in MSFsc. The posthoc‐analyses indicate that the findings of the main GEE analyses should be mainly attributed to the subgroup of participants that advanced in their chronotype over time.

**Table 3:** Results of the GEE post‐hoc analyses: longitudinal associations between change in severity of depressive and anxiety symptoms and change in chronotype (MSFsc) analyzed by generalized estimating equations per stability chronotype group (advanced, stable, and delayed)

	MSFsc								
	Advanced (*N* = 473)			Stable (*N* = 665)			Delayed (*N* = 279)		
	B	95% CI	*p*	B	95% CI	*p*	B	95% CI	*p*
Model 1									
Depressive symptoms	0.009	0.002–0.016	**.010**	0.000	−0.002–0.003	.764	0.003	−0.006–0.013	.467
Model 2									
Anxiety symptoms	0.012	0.002–0.021	**.017**	0.000	−0.004–0.003	.859	0.002	−0.011–0.014	.778
Model 3									
Depressive symptoms	0.006	−0.005–0.017	.286	0.001	−0.002–0.004	.587	0.006	−0.008–0.019	.403
Anxiety symptoms	0.006	−0.009–0.021	.437	−0.001	−0.005–0.003	.627	−0.004	−0.022–0.014	.675
Model 4^a^									
Depressive symptoms	0.014	0.003–0.025	**.012**	0.001	−0.010– −0.001	.489	0.004	−0.009–0.018	.545
Anxiety symptoms	0.003	−0.011–0.016	.712	−0.001	−0.005–0.003	.663	−0.001	−0.018–0.017	.945
Model 5^b^									
Depressive symptoms	0.012	0.002–0.022	**.015**	0.001	−0.002–0.004	.432	0.007	−0.006–0.019	.314
Anxiety symptoms	0.004	−0.009–0.017	.532	−0.001	−0.005–0.003	.592	−0.002	−0.017–0.014	.814

Abbreviations: CI, confidence interval; GEE, generalized estimating equation; MSFsc, MidSleep on free days sleep corrected.

^a^ Model 4: Additionally adjusted for sex, children in household, employment, insomnia level.

^b^ Model 5: Additionally adjusted for age.

## 4 DISCUSSION

This paper shows chronotype to be stable over a 7‐year follow‐up period even though it had become 10.8 min earlier on average. These findings indicate that even though chronotype is stable at group level (i.e., an individual's ranking within the sample), chronotype can change on an individual level. A similar construct is found in longitudinal analyses of personality traits, where a high test–retest correlation can also co‐occur with a difference in mean level (Ormel et al., 2013; Roberts, Walton, & Viechtbauer, 2006; Srivastava, John, Gosling, & Potter, 2003). This could be a result of a combination of factors, such as genetic factors (Toomey, Panizzon, Kremen, Franz, & Lyons, 2015), which are generally stable over time, and time‐specific environmental factors, for example, change in sleep timing because of work or retirement.

Furthermore, both a decrease in severity of depressive symptoms and anxiety symptoms were associated with an advance in chronotype. Yet, when analyzed in multivariate models, only a decrease in severity of depressive symptoms was found to be robustly associated with an advance in chronotype. These results were confirmed in our posthoc‐analyses. As outlined in the introduction, previous literature suggested chronotype to be a trait associated with vulnerability of developing a depressive disorder (Drennan et al., 1991; Merikanto et al., 2013). The concordant change in depressive symptoms severity and chronotype that we found, confirms a close relationship between these two constructs. Together with our finding that chronotype is stable over 7 years, we conclude that chronotype should be considered as a mostly trait‐like construct that is associated with current mood and may change over time. However, firmly classifying chronotype as either a trait or state construct is difficult based on our findings. in particular as longitudinal stabilities of state and trait constructs are in general more comparable than usually assumed as is explained in the review of Ormel et al. (2013).

Based on findings from a cross‐sectional study where chronotype advanced with age (age range 10–80 years; Roenneberg et al., 2007), the change in chronotype in our study could be due to normative aging of the participants over the follow‐up period. However, our sample had an age range of 42.46 (*SD*, 12.78) at T1 and 49.54 (*SD*, 12.79) at T2 and thus mainly consists of respondents in their middle ages, an age range not associated with showing a large change in chronotype according to the results of Roenneberg et al. (2007). Unfortunately, firm developmental conclusion cannot be drawn from such cross‐sectional data. We can state that in our own analyses, controlling for age did not change our main findings. We therefore conclude that aging was not the key effector of the change in chronotype in our sample. Alternatively, the concordant change in depressive symptoms and chronotype may be explained by a changing sleep pattern associated with developing depressive symptoms. Sleep related variables, such as shorter and longer sleep durations, as well as sleeping difficulties, are found to be predictive of a chronic course of depressive and anxiety disorder (Luik et al., 2015; van Mill, Vogelzangs, van Someren, Hoogendijk, & Penninx, 2014). It is possible that chronotype changes as a result of the changing sleep pattern and this in turn is associated with changing depressive symptoms. Monitoring sleep duration and changes in chronotype might therefore be a way to complement the current clinical evaluation of persons suffering from depressive symptoms.

Both a change in severity of anxiety symptoms and depressive symptoms were associated with a change in chronotype when analyzed as a single variable entered in the analysis. This might be a result of the high correlation between these two severity variables, which is not surprising considering the high comorbidity between anxiety and depressive disorders (Hirschfeld, 2001). According to the cut‐off score that was chosen, there was no multicollinearity (Field, 2009). Yet, it remains an arbitrary cut‐off score and moderate multicollinearity may still have affected these analyses. However, when both severity measures were entered simultaneously in the model, there is no association between a change in severity of anxiety symptoms and a change in chronotype, which is noteworthy. Looking at the *b*‐values of the GEE analyses in Model 3–5 (table 2), the B‐values of depressive symptoms are very similar in the different models, while the B‐values of anxiety symptoms are lower when depressive symptoms are included in the models as well. Consistent with our findings, a previous review also reports on mixed findings on the relationship between anxiety symptoms and chronotype (Kivelä, Papadopoulos, & Antypa, 2018). Following the theory of the tripartite model (Clark & Watson, 1991), it is tempting to speculate that these results can be interpreted as that chronotype is associated with the shared part of anxiety and depression (i.e., the negative affect component), and incrementally with variance specific to depression (low positive affect, or anhedonia), but not the specific component of anxiety (hyperarousal). However, more research is needed to give a conclusive answer to this question. In our study, the level of severity of depressive symptoms was not different between T1 and T2 in the total sample, as well as in the delayed, stable and advanced chronotype groups separately. This is an interesting finding as the prevalence of depressive disorder diagnoses did decrease over this period. It should however be noted that the number of patients with a current depressive disorder diagnosis was low both at T1 and T2 (12.63% and 9.81%, respectively) and did not affect the mean level of depressive symptoms. The fact that mean level of depressive symptoms is relatively high (13.80, *SD*, 10.82 at T1) can be explained by the fact that the majority of participants that were included at the baseline of NESDA had a current or lifetime diagnosis of a depressive or anxiety disorder (Penninx et al., 2008).

When interpreting the results of this study, the following strengths and limitations should be considered. An important strength is the large sample size that was used for the analyses. Additionally, to our knowledge, this is the first study that was able to test the longitudinal stability of chronotype calculated from reported actual sleep timing (MSFsc, assessed by the MCTQ) contrary to an individuals preferred sleep timing (Broms et al., 2014; Caci et al., 2000; Koskenvuo et al., 2007). Chronotype from actual sleep times correlates highly with dim light melatonin onset, which is the golden standard to estimate the circadian timing in humans, and should therefore be considered a reliable measurement of someone's chronotype (Kantermann, Sung, & Burgess, 2015). However, because the MCTQ uses actual sleep timing, the outcome is affected by factors, such as worktimes and having children in one's household. Therefore, the MCTQ might be less stable than questionnaires assessing preference of sleep timing (e.g., MEQ). It remains to be seen in future research whether using chronotype measures with preferred sleep timing yield similar results as are shown here. Another limitation of the study is the small change in the chronotype questions in the two waves. We examined plausible methods for calculating chronotype and repeated all analyses as a robustness check. As there were no differences between these results, the difference in questionnaires did not cause differences in results and thus interpretation of these. Finally, only two repeated measurements were available in our sample. A more accurate insight could possibly have been obtained with more repeated measurements.

To conclude, chronotype was found to be a stable trait‐like construct with only a minor level advance (i.e., chronotype became earlier) over a period of 7 years. Changes in chronotype were in concord with changes in severity of depressive, but not anxiety, symptoms.

## CONFLICT OF INTERESTS

The authors declare that there are no conflict of interests.

## Supporting information

Supporting information

## Data Availability

The data that support the findings of this study are available from the Netherlands Study of Depression and Anxiety. Restrictions apply to the availability of these data, which were used under licence for this study.
