# Supplementary material for: Stability of chronotype over a 7‐year follow‐up period and its association with severity of depressive and anxiety symptoms
Source: Depress Anxiety. 2020 Feb 17;37(5):466–74. doi: 10.1002/da.22995 (PMC7318352; doi:10.1002/da.22995)
Supplement: Supplementary file 1 — Supporting information [file DA-37-466-s001.docx]

**SUPPLEMENTAL MATERIAL**

**Table S1:** Spearman Rank correlation coefficients between covariates (N=1417)

|  | Depressive symptoms | Anxiety symptoms | Insomnia | Sex | Age | Children in household | Employment |
| --- | --- | --- | --- | --- | --- | --- | --- |
| Depressive symptoms | ---- | 0.788** | 0.494** | 0.063* | 0.059* | -0.025 | -0.162** |
| Anxiety symptoms |  | ---- | 0.387** | 0.080** | 0.052 | -0.065* | -0.151** |
| Insomnia |  |  | ---- | 0.062* | 0.225** | -0.029 | -0.060* |
| Sex |  |  |  | ---- | -0.106** | 0.040 | -0.024 |
| Age |  |  |  |  | ---- | 0.089** | 0.001 |
| Children in household |  |  |  |  |  | ---- | 0.140** |
| Employment |  |  |  |  |  |  | ---- |
| Note: ** Correlation is significant at the 0.01 level; * correlation is significant at the 0.05 level. | | | | | | | |

**Chronotype calculation at T1 and T2**

Chronotype, MSFsc, is calculated as the midpoint of sleep onset and offset, corrected by subtracting from MSF half of the difference between sleep duration on free days and average weekly sleep duration (Roenneberg, Allebrandt, Merrow, & Vetter, 2012).

Sleep onset at T1 was assessed by the questions:

- ‘I go to bed at ..’
- ‘Minutes needed to fall asleep’

Sleep onset at T2 was assessed by the questions:

- ‘I go to bed at ..’
- ‘I decide to go to sleep at ...’
- ‘Minutes needed to fall asleep’

MSFsc in the main analyses was calculated using the latest time of the two answers to ‘I go to bed at’ and ‘I decide to go to sleep at ..’. For the additional analyses (table S2 – S3) MSFsc is calculated with using sleep onset as:

- ‘I go to bed’ + ‘Minutes needed to fall asleep’ (MSFsc_BT)
- ‘I decide to go to sleep at ...’ + ‘Minutes needed to fall asleep’ (MSFsc_ST)

Depending on the data that were available from these questions, sample size differed slightly between MSFsc (N=1417), MSFsc_BT (N=1420) and MSFsc_ST (N=1410).

**Table S2:** Additional analyses of the longitudinal associations between change in severity of depressive and anxiety symptoms and change in chronotype analyzed by generalized estimating equations (n=1420)

|  | MSFsc_BT ^a^ | | |
| --- | --- | --- | --- |
| Main predictor | B | 95% CI | P |
| *Model 1* |  | | |
| Depressive symptoms | 0.005 | 0.001 – 0.009 | **0.018** |
| *Model 2* |  | | |
| Anxiety symptoms | 0.007 | 0.001 – 0.012 | **0.019** |
| *Model 3* |  | | |
| Depressive symptoms | 0.003 | -0.003 – 0.008 | 0.366 |
| Anxiety symptoms | 0.004 | -0.004 – 0.012 | 0.343 |
| *Model 4 ^b^* |  | | |
| Depressive symptoms | 0.006 | 0.001 – 0.012 | **0.028** |
| Anxiety symptoms | 0.004 | -0.004 – 0.011 | 0.361 |
| *Model 5 ^c^* |  | | |
| Depressive symptoms | 0.006 | 0.001 – 0.012 | **0.025** |
| Anxiety symptoms | 0.002 | -0.005 – 0.010 | 0.548 |
| Note: ^a^  MSFsc_BT: MidSleep on Free days sleep corrected, as explained in the text above; ^b^ Model 4: additionally adjusted for sex, children in household, employment, insomnia level; ^c^ Model 5: additionally adjusted for age | | | |

**Table S3:** Additional analyses of the longitudinal associations between change in severity of depressive and anxiety symptoms and change in chronotype (MSFsc_ST) analyzed by generalized estimating equations (N=1410).

|  | MSFsc_ST ^a^ | | |
| --- | --- | --- | --- |
| Main predictor | B | 95% CI | P |
| *Model 1* |  | | |
| Depressive symptoms | 0.005 | 0.002 – 0.009 | **0.005** |
| *Model 2* |  | | |
| Anxiety symptoms | 0.006 | 0.000 – 0.011 | **0.033** |
| *Model 3* |  | | |
| Depressive symptoms | 0.005 | 0.000 – 0.010 | 0.071 |
| Anxiety symptoms | 0.001 | -0.007 – 0.009 | 0.801 |
| *Model 4 ^b^* |  | | |
| Depressive symptoms | 0.008 | 0.002 – 0.013 | **0.005** |
| Anxiety symptoms | 0.000 | -0.007 – 0.008 | 0.905 |
| *Model 5 ^c^* |  | | |
| Depressive symptoms | 0.008 | 0.002 – 0.013 | **0.004** |
| Anxiety symptoms | 0.000 | -0.008 – 0.007 | 0.933 |
| Note: ^a^  MSFsc_ST: MidSleep on Free days sleep corrected, as explained in the text above; ^b^ Model 4: additionally adjusted for sex, children in household, employment, insomnia level; ^c^ Model 5: additionally adjusted for age | | | |


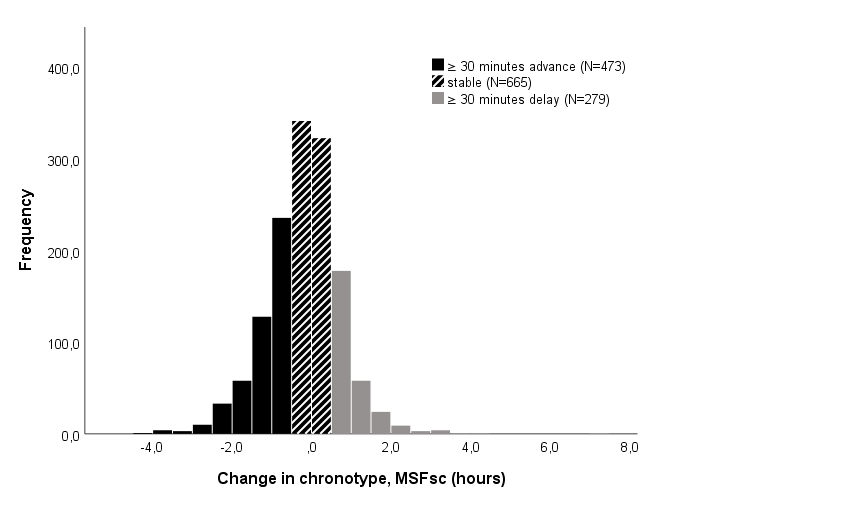


**Figure S1**: Histogram of the change in chronotype (= MSFsc T2 – MSFsc T1), between T1 and T2 (N=1417)

**Table S4:** Sociodemographic, lifestyle and clinical factors compared over seven years per stability chronotype group (advanced, stable and delayed)

| **Advanced (N=473)** | | | |
| --- | --- | --- | --- |
| Characteristics | T1 | T2 | P^a^ |
| Sex (n (%), female) | 320 (67.70) | 320 (67.70) | - |
| Age (in yrs, M (SD)) | 37.46 (12.70) | 44.57 (12.71) | **<0.001** |
| Child in household (n (%), yes) | 108 (22.80) | 213 (45.00) | **<0.001** |
| Employment status (n (%), yes) | 344 (72.7) | 354 (74.8) | 0.723 |
| Depressive symptoms (M (SD)) | 13.79 (10.92) | 13.25 (10.63) | 0.092 |
| Anxiety symptoms (M (SD)) | 7.72 (7.91) | 7.26 (8.23) | **0.028** |
| Insomnia (M (SD)) | 6.24 (4.24) | 6.88 (4.59) | **0.008** |
| Depressive disorder diagnosis CIDI^b^ (n (%), yes) | 66 (14.00) | 43 (9.10) | **0.013** |
| Anxiety disorder diagnosis CIDI^b^ (n (%), yes) | 91 (19.20) | 61 (12.90) | **0.002** |
| Chronotype in MSFsc (M (SD)) | 4.57 (1.00) | 3.40 (0.91) | **<0.001** |
| **Stable (N=665)** | | | |
|  | T1 | T2 | P^a^ |
| Sex (n (%), female) | 430 (64.70) | 430 (64.70) | - |
| Age (in yrs, M (SD)) | 44.53 (12.10) | 51.61 (12.11) | **<0.001** |
| Child in household (n (%), yes) | 272 (40.90) | 248 (37.30) | **0.044** |
| Employment status (n (%), yes) | 530 (79.70) | 470 (70.70) | **<0.001** |
| Depressive symptoms (M (SD)) | 12.56 (9.84) | 13.03 (10.52) | 0.592 |
| Anxiety symptoms (M (SD)) | 6.57 (6.80) | 6.47 (6.70) | 0.562 |
| Insomnia (M (SD)) | 6.77 (4.33) | 6.88 (4.51) | 0.753 |
| Depressive disorder diagnosis CIDI^b^ (n (%), yes) | 67 (10.10) | 66 (9.90) | 1.000 |
| Anxiety disorder diagnosis CIDI^b^ (n (%), yes) | 111 (16.70) | 95 (14.30) | 0.174 |
| Chronotype in MSFsc (M (SD)) | 3.72 (0.73) | 3.71 (0.75) | 0.638 |
| **Delay (N=279)** | | | |
|  | T1 | T2 | P^a^ |
| Sex (n (%), female) | 185 (66.30) | 185 (66.30) | - |
| Age (in yrs, M (SD)) | 45.99 (11.98) | 53.04 (11.99) | **<0.001** |
| Child in household (n (%), yes) | 107 (38.40) | 81 (29.00) | **<0.001** |
| Employment status (n (%), yes) | 210 (75.30) | 174 (62.40) | **<0.001** |
| Depressive symptoms (M (SD)) | 16.46 (12.40) | 16.07 (11.77) | 0.424 |
| Anxiety symptoms (M (SD)) | 8.96 (9.30) | 8.06 (8.87) | **0.033** |
| Insomnia (M (SD)) | 7.77 (4.81) | 7.00 (4.70) | **0.006** |
| Depressive disorder diagnosis CIDI^b^ (n (%), yes) | 46 (16.50) | 30 (10.80) | **0.027** |
| Anxiety disorder diagnosis CIDI^b^ (n (%), yes) | 61 (21.90) | 44 (15.80) | **0.022** |
| Chronotype in MSFsc (M (SD)) | 3.48 (0.91) | 4.52 (0.08) | **<0.001** |
| Note: ^a^ Wilcoxon signed rank tests were used to compare the continuous characteristics (age, depressive symptoms, anxiety symptoms, insomnia symptoms), McNemar tests were used to compare dichotomous characteristics (child in household, employment status, depressive disorder diagnosis, anxiety disorder diagnosis); ^b^ The one-month CIDI diagnoses were used (diagnosis present in the month prior to the assessment); T1: NESDA’s 2-year follow-up, T2: NESDA’s 9-year follow-up, MSFsc: MidSleep on Free days sleep corrected, CIDI: Composite International Diagnostic Interview | | | |
